# The Integration of Epistasis Network and Functional Interactions in a GWAS Implicates RXR Pathway Genes in the Immune Response to Smallpox Vaccine

**DOI:** 10.1371/journal.pone.0158016

**Published:** 2016-08-11

**Authors:** Brett A. McKinney, Caleb Lareau, Ann L. Oberg, Richard B. Kennedy, Inna G. Ovsyannikova, Gregory A. Poland

**Affiliations:** 1 Tandy School of Computer Science and Department of Mathematics, University of Tulsa, Tulsa, OK, United States of America; 2 Division of Biomedical Statistics and Informatics, Department of Health Sciences Research, Mayo Clinic, Rochester, MN, United States of America; 3 Mayo Clinic Vaccine Research Group, Mayo Clinic, Rochester, MN, United States of America; Case Western Reserve University, UNITED STATES

## Abstract

Although many diseases and traits show large heritability, few genetic variants have been found to strongly separate phenotype groups by genotype. Complex regulatory networks of variants and expression of multiple genes lead to small individual-variant effects and difficulty replicating the effect of any single variant in an affected pathway. Interaction network modeling of GWAS identifies effects ignored by univariate models, but population differences may still cause specific genes to not replicate. Integrative network models may help detect indirect effects of variants in the underlying biological pathway. In this study, we used gene-level functional interaction information from the Integrative Multi-species Prediction (IMP) tool to reveal important genes associated with a complex phenotype through evidence from epistasis networks and pathway enrichment. We test this method for augmenting variant-based network analyses with functional interactions by applying it to a smallpox vaccine immune response GWAS. The integrative analysis spotlights the role of genes related to retinoid X receptor alpha (*RXRA*), which has been implicated in a previous epistasis network analysis of smallpox vaccine.

## Introduction

In a functional gene network approach, Franke *et al*. [1] demonstrated that genes for multigenic disorders tend to be in similar functional pathways and these genes tend to have more functional interactions. In their biofilter approach for prioritizing genes for GWAS analysis, Bush *et al*. [2] point out that epistasis may lead to a failure of replication of single-SNP effects. Specifically, the effect of one allele may depend on the presence of a second unknown allele, and if the unknown allele is not present in a new population, the candidate effect will not replicate. Thus, specific associations may not replicate in a new population due to epistasis, but associations and interactions among other SNPs are likely to be observed in genes from common pathways. We hypothesize that looking for evidence of coordinated activity of a pathway from a set of variants prioritized by epistatic effects, as well as main effects, may permit one to predict the role of genes that might otherwise be false negatives.

Recently, Greene *et al*. [3] combined weak main effects with functional interaction networks from the Integrated Multi-species Prediction (IMP) database and showed that the top variants participate in functional genesets. This result suggests that complex genetics of multigenic phenotypes may be inferred by pairing weak main effects with *a priori* functional information. In the current study, instead of using main effects only, we prioritize by main effect and epistasis network centrality. In addition, we use pathway enrichment and IMP to further characterize the functional network of complex phenotypes.

The integrative component of the current approach builds upon our previous feature-selection algorithms that incorporate main and interaction effects: simulated evaporative cooling (SEC) [4], regression model-based genetic association interaction networks (reGAIN) [5], and an epistasis network centrality (SNPrank) [6]. We have applied these filtering methods in the context of pathway analysis by replicating enriched pathways in GWAS of bipolar disorder [7]. Another related approach used an L1 pathway regularization to identify important gene-gene interactions [8]. We further review these filtering algorithms in the Methods section.

The focus of the applied analysis in the current study is a GWAS of the human immune response to smallpox vaccination. A previous analysis in a different population implicated a variant (rs1805352) in retinoid X receptor alpha (*RXRA*) using a gene network centrality algorithm in immune response to smallpox vaccine [6]. In that study, an EC filtered epistasis network (reGAIN) was analyzed by SNPrank to determine that the RXRA variant showed a strong centrality for smallpox vaccine immune response. This importance of RXRA using an epistasis network framework has been supported by traditional approaches that implicate variants in the *RXRA* gene in immune response to hepatitis B, measles, and rubella vaccines [9,10,11]. Thus, although this previous study had a smaller sample size, a gene interaction network framework enabled the identification of biologically relevant effects.

In the current study, we expand the machine learning filtering and gene interaction network analysis to include an integrative framework to fill gaps in GWAS data that may occur due to allele frequency differences between study populations or, as in the current study, when there is not a strong tag for a SNP of interest. After filtering, we used pathway enrichment to prioritize genes from the epistasis network that are likely involved in the same functional pathway, and hence share involvement in response to smallpox vaccination. We used the genes that were prioritized by filtering and that were in the most significant pathway as input to IMP to predict a functional interaction network. The functional interaction network showed a physical interaction between one of the input genes (THRB) and RXRA, and the functional network showed an enrichment of RXR and retinoic acid pathways. The integration of multiple levels of information provides a more comprehensive view of genes and pathways likely to be involved in the vaccine immune response phenotype.

## Methods

### Subjects and Data

Subjects from a previously described smallpox vaccine cohort (18–40 years old) were utilized for this study [1,12]. Study subjects had been vaccinated with a single dose of smallpox vaccine **(Dryvax, Wyeth Laboratories)** between 2002 and 2006. All subjects had a documented vaccine “take” at the vaccination site. Smallpox antibody response GWAS data collected at Mayo Clinic (Rochester, MN) [12,13] included genotype and phenotype information for 1,000 individuals of varying race and gender (732 males, 268 females) and 455,986 SNPs. To reduce confounding, individuals were filtered by race to European Ancestry (EA). After filtering, there were 523 individuals remaining (383 males, 140 females). The Institutional Review Board of Mayo Clinic approved the study, and written informed consent was obtained from each subject.

### Neutralizing Antibody Measurement

Vaccinia-specific neutralizing antibody (NA) titers were quantified for each subject using a method that has been previously published by us [14]. Measurements were reported as the serum dilution that inhibits 50% of virus activity (ID50). The coefficient of variation for NA assay was 6.9%.

### Integrative Epistasis Network Approach

**Fig 1:**
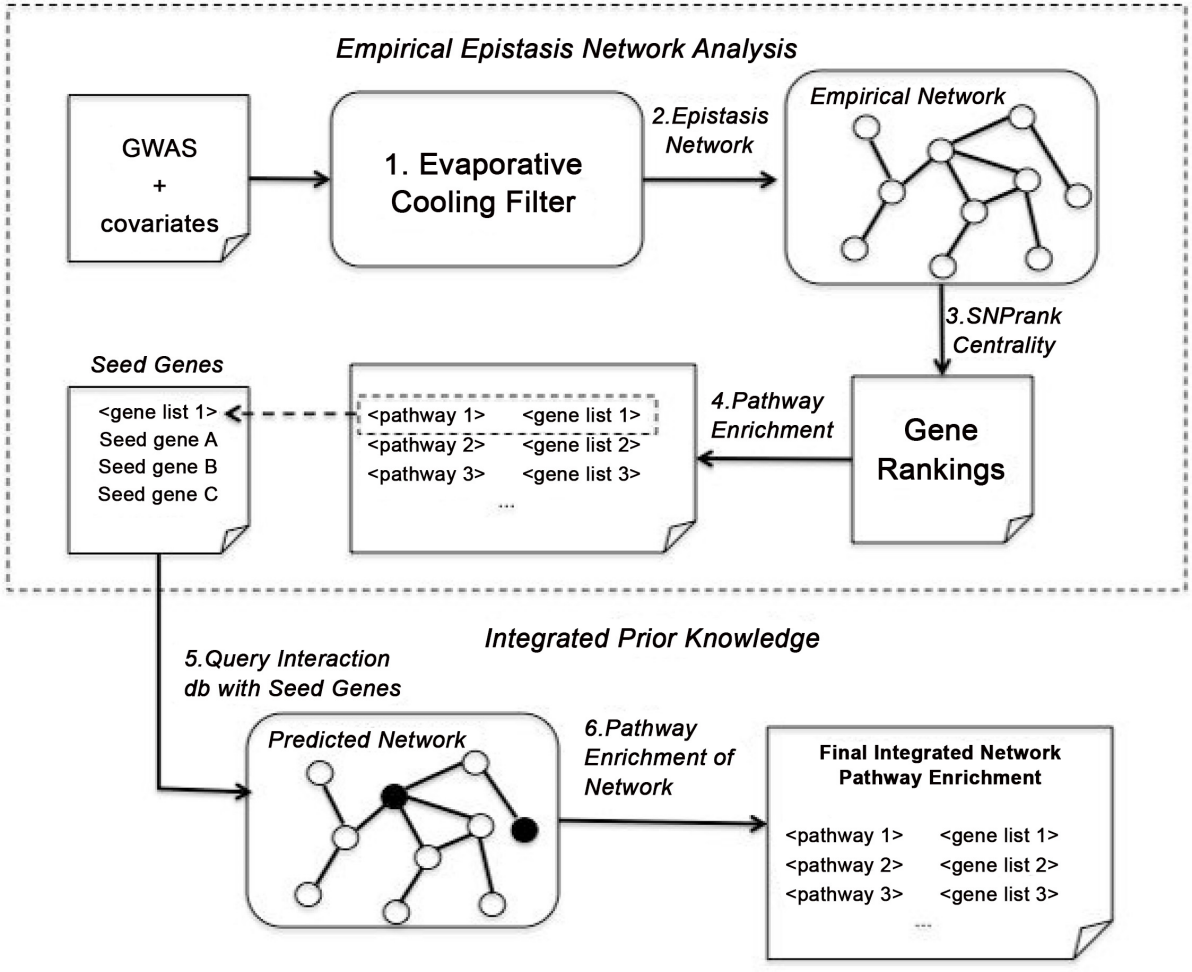
Integrative epistasis network analysis strategy. Simulated evaporative cooling (EC) feature selection is used to filter the top SNPs (1), which are used to compute a regression genetic association interaction network (reGAIN) of epistatic and main effects (2). The most important (hub) SNPs from the reGAIN are identified by their centrality with SNPrank (3). The top SNPs are mapped to genes and used to determine the most enriched Reactome pathways (4). The genes from the top pathway are used to query IMP for known functional interactions. Filled circles represent query genes and open circles represent predicted genes based on prior functional connectivity (5). The output is a posterior functional interaction network with enriched pathways (6).

Our bioinformatics strategy (Fig 1) integrates empirical epistasis network calculations with predicted network information to identify biologically relevant pathways in the GWAS of smallpox immune response. While previous epistasis gene network approaches often utilize only phenotype-specific information (steps 1–4), we sought to determine pathways from a full source of prior biological knowledge utilizing the phenotype-specific genes as “seeds.” Details of the analysis strategy are as follows.

#### Step 1. Machine-learning variant filtering protocol

Simulated Evaporative Cooling (EC) is a machine-learning algorithm that incorporates main effect contributions with interaction effects to prioritize variants [4,15]. The algorithm removes the least important SNPs in an iterative process analogous to physical cooling of a gas by evaporation of atoms. Part of the EC simulation process involves balancing the main effect and interaction contribution to each predictor’s score. Whereas standard linear regression for variant prioritization ignores interaction effects, EC uses ReliefF to calculate the interaction component for each SNP, providing a more robust indication of a variant’s utility in a posterior epistatic network. The other half of EC is Random Jungle, an implementation of Random Forest, which is used to calculate the main effect contribution of the SNP importance scores. By filtering variants before the construction of an epistasis network, we significantly reduce the computational burden of computing pairwise interactions in Step 2 without sacrificing variants likely to be integral in an epistasis network. One could use a univariate filter instead, but as mentioned in the introduction, we hypothesize that including interactions will be important for characterizing the full pathway for a phenotype.

#### Step 2. Computation of epistasis network

Many of the interactions and main effects are already captured in Step 1, so one could set a threshold for the EC scores and skip to Step 4. However, Steps 2 and 3 help further filter noise variants and construct an epistasis network that may be integrated with other network information. From the top 1,000 EC-filtered SNPs, we used the generalized linear model (GLM) to calculate the pairwise interaction strengths and main effects. We use these regression model coefficients to construct a regression GAIN (reGAIN) matrix [5], which is a form of epistasis network. We restricted the number of variants used in this step to 1,000 in order minimize the computational burden associated with computing the pairwise interactions. Using the antibody titers as the outcome (phenotype), individual variants and multiplicative interaction terms were used as predictors. Covariates included sex, age, quartile of years from immunization to blood draw, season, and a binary variable to indicate ambient versus frozen shipping temperature. The standardized coefficients from the regression models encode a reGAIN matrix with each SNP’s main effect coefficient along the diagonal and SNP-SNP interaction coefficients on the off-diagonals. The resulting symmetric matrix encodes a network of variants (nodes) and their statistical interactions (edges) based on the GLM.

#### Step 3. Variant prioritization using a network framework

Using the reGAIN matrix from Step 2, we employed an eigenvector centrality algorithm called SNPrank [5] to prioritize the variants whose aggregate interactions and main effects contributed most to the variance in the antibody titer. This additional interaction and main-effect filtering step removes more irrelevant SNPs while adjusting for covariates. To assess the possible biological activity of the variants implicated in our epistasis network, variants with the strongest centrality were mapped to their corresponding genes and used for enrichment analysis.

#### Step 4. Pathway Enrichment from empirical epistasis network

To characterize the pathways and processes associated with the most central genes in our epistasis network, we used the Reactome Functional Interaction (FI) database to identify enriched biological pathways in the top genes [16]. As we previously restricted the number of variants used to construct our epistasis network, we used the top 200 genes based on the epistasis network SNPrank centrality analysis of the GWAS as input to Reactome pathway enrichment. The choice of 200 genes is data dependent, but is generally a reasonable number for enrichment [17]. In the Methods section, we further discuss this choice of number of genes.

#### Step 5. Functional interaction prediction from data-driven analysis

The epistasis network centrality reflects main effects and statistical interactions from the GWAS data, and the prioritization of genes by pathway enrichment reflects their coordinated effect on the phenotype. To identify additional potential interaction partners and relevant genes, we queried IMP, which integrates multiple sources of evidence for functional interactions [18]. We used the data-driven prioritized genes in the most significantly enriched pathway as seeds for predicting functional interactions. We queried IMP with the pathway-enriched genes, as opposed to a larger list, because the enrichment suggests coordinated activity of the genes and, hence, good candidates for finding interactions that might have been missed in the data-driven analysis.

#### Step 6. Functional interaction network enrichment

After integrating our GWAS-driven genes from the statistical epistasis network with prior functional interactions from IMP (Step 5), we next identified pathway enrichment present in the posterior functional interaction network. The posterior functional interaction network from IMP includes additional predicted genes and interactions. Unlike the enrichment in Step 4, which reflected biological pathways strictly from factors present in the phenotype-specific results, the resulting biological pathways from Step 6 represent biological pathways enriched when integrating prior biological knowledge with the enriched processes responding to smallpox vaccine response.

## Results

**Fig 2:**
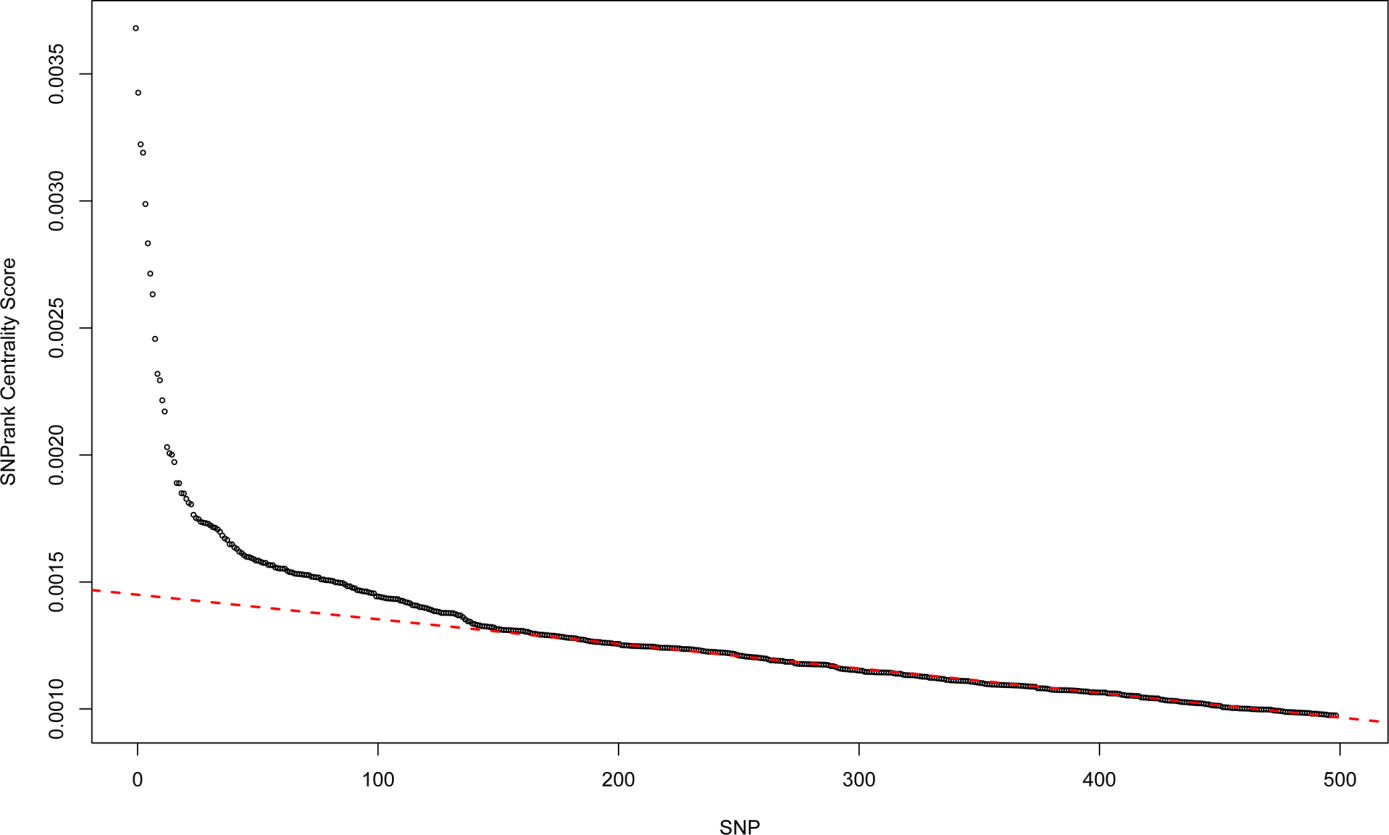
SNPrank centrality score elbow plot. The SNPrank scores are plotted for the top 500 variants. The red dashed line represents the null centrality line.

After EC filtering (Fig 1, Step 1 of integrative strategy) and regression epistasis network analysis adjusted for covariates (Fig 1, Step 2), we reprioritized the genes in the reGAIN using SNPrank centrality to remove additional noise variants (Fig 1, Step 3). For pathway enrichment (Fig 1, Step 4), we selected the top 200 SNPrank genes. The elbow plot of the SNPrank scores (Fig 2) shows that above 200 SNPs, the scores appear consistent with a null centrality. Based on the separation of the elbow from the null line, one could make an argument for using a smaller number of genes in the pathway enrichment, but another motivation for selecting the threshold number of genes for enrichment is to obtain a large enough collection of genes to detect relevant pathways. The choice of number of genes is data dependent, but generally, 200 is reasonable for enrichment [17]. For example, the immunologic genesets (C7) in MSigDB were constructed from the 200 top or bottom genes (or FDR < 0.25) for comparisons between conditions in immunologic gene expression experiments [19].

**Fig 3:**
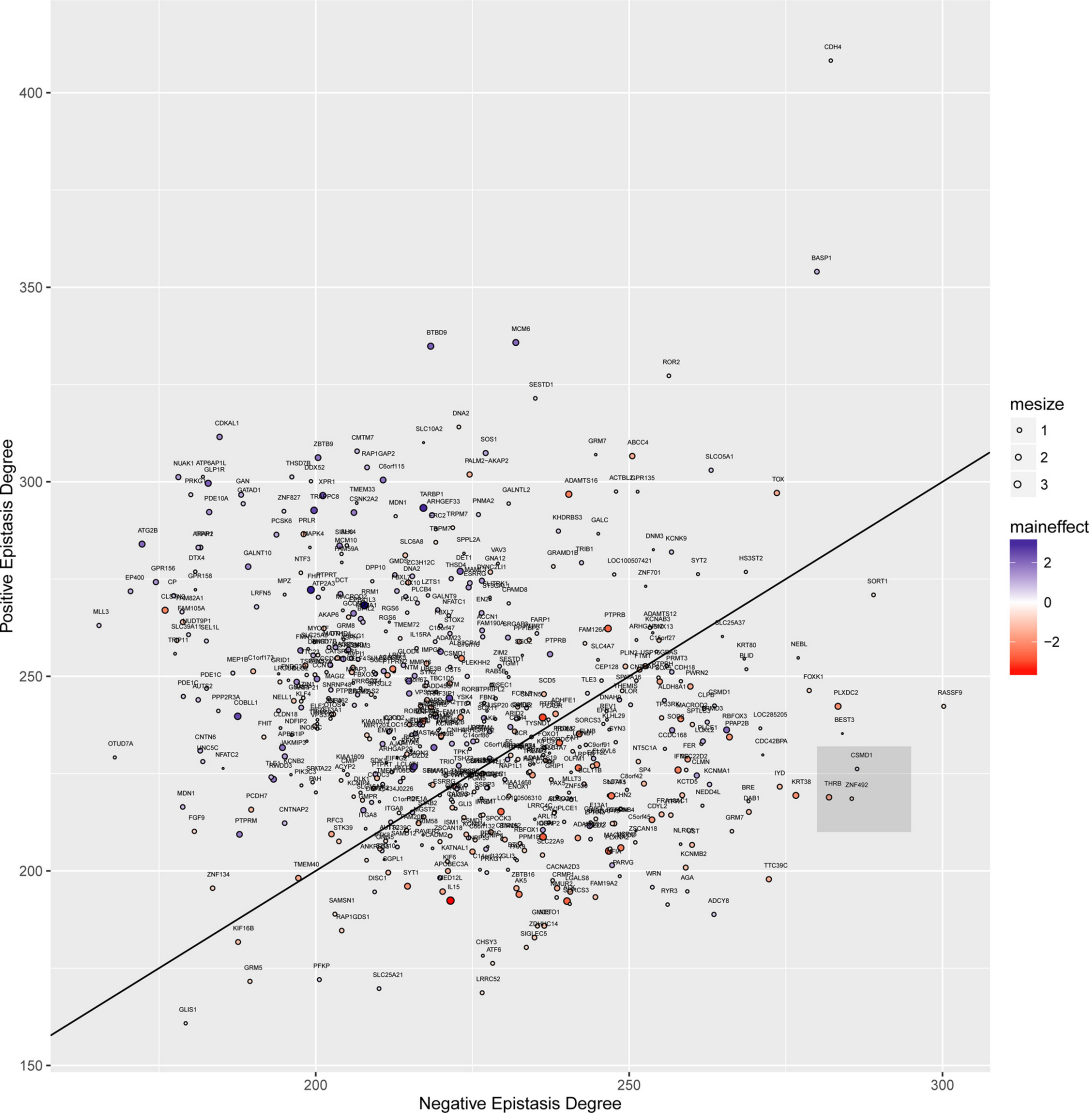
Positive/negative epistasis degree plot shows the overall epistatic network effect and main effect of the top variants for smallpox vaccine immune response. For each variant (mapped to gene symbol), the sum of positive interaction coefficients (positive epistasis degree) versus negative epistasis degree is plotted. The diagonal is the line of zero sum of epistasis degree. Plot symbols (size and color) are labeled by their main effect (magnitude and direction of effect on vaccine immune response). The gray box highlights the *THBR* variant.

The epistasis network effects and main effects of the top SNPrank genes can be visualized in the “Positive/Negative Epistasis Degree Plot” (Fig 3), which we previously developed for differential co-expression network visualization [20]. Positive (negative) epistasis degree is the sum of all positive (negative) epistasic interaction coefficients for the given variant in the epistasis network. Positive (negative) epistasis in this context means an interaction that leads to higher (lower) vaccine immune response. The vertical (horizontal) coordinate of each point is the sum of the positive (negative) interactions. Variants with a greater number of interactions that lead to lower immune response fall below the diagonal line. The points are also labeled by their main effect (blue gradient for association with higher immune response and red gradient for association with lower immune response). Variants with negative epistatic effects (below diagonal) also tend to have negative main effects (red). In the gray box, THRB, for example, has a large number of negative epistatic effects (below diagonal) and a weak negative main effect. IL15 (large red point) is the largest negative main effect (susceptibility to low response) in the filtered data. We note that interactions are essential for finding the important genes THRB and SOS1; they are not in the top 1000 by a univariate analysis.

The top 200 variants from the SNPrank centrality analysis were mapped to corresponding genes based on variant proximity to the gene body and analyzed to infer pathway enrichment from the Reactome database. The top pathway “Map kinase inactivation of SMRT corepressor (B)” (p = 0.0002, fdr = 1.42e-01) contains epistasis network genes *ZBTB16*, *SOS1*, and *THRB*. Phosphorylation causes SMRT (silencing mediator of retinoic acid and thyroid hormone receptor) to unbind from transcription factor complexes like RXR and RAR. In this top pathway, the gene *ZBTB16* (zinc finger and BTB domain containing 16), which also goes by the name *PLZF* (promyelocytic leukemia zinc finger), has been shown to regulate the transcriptional activity of Retinoic Acid Receptor (RARs) transcriptional activity [21]. The involvement of *ZBTB16* with retinoid receptors is also suggested by the association of a chromosomal translocation to a rare variant of acute promyelocytic leukemia (APL), which fuses the *ZBTB16* (*PLZF*) protein to retinoic acid receptor (*RARA*) [22]. The gene *THRB* (thyroid hormone receptor, beta) in the pathway has a physical interaction with *RXRA* [23].

**Fig 4:**
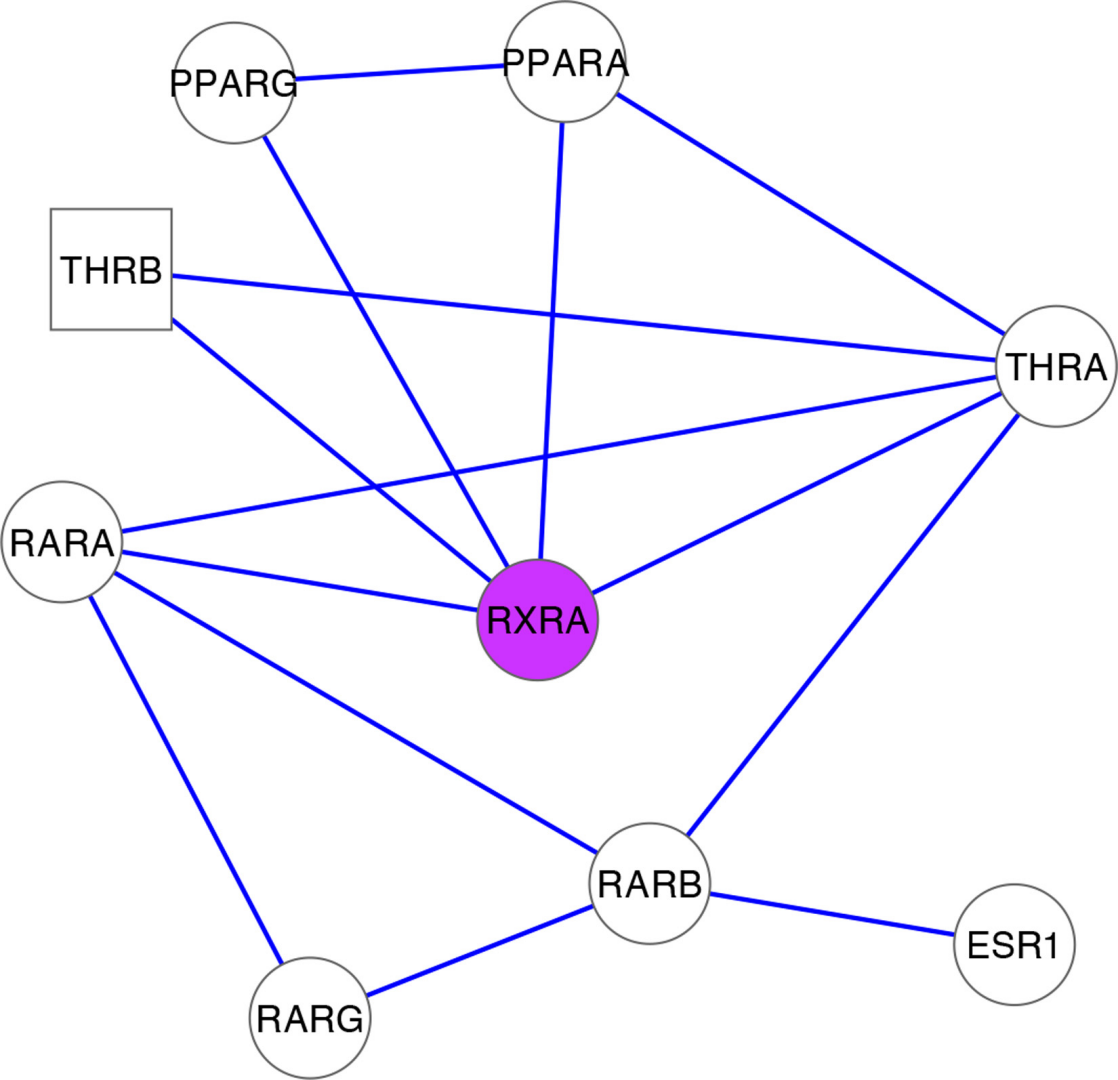
RXR network predicted by IMP using *THRB* (square node) as a seed from the empirical epistasis network analysis. Empirical seed was chosen from the top enriched pathway from the epistasis network analysis of the smallpox vaccine GWAS. Variants in *RXRA* (purple node) have been previously associated with variation in smallpox vaccination response using an epistasis network centrality approach.

In the epistasis network analysis, genes such as *ZBTB16* and *THRB* in the top pathway suggest the involvement of RXR pathways; however, as mentioned previously, the particular *RXRA* SNP variant was not genotyped in the current GWAS. In order to further characterize the involvement of RXR genes and other genes involved in smallpox vaccine immune response, we used the genes in the most enriched epistasis network pathways as seeds for predicting an interaction network from integrated prior knowledge (Step 5, Fig 1). For this purpose, we queried the IMP portal [18], which integrates multiple sources of evidence to predict new network interaction partners (Fig 4).

The edges in the predicted network (Fig 4) are weighted by the posterior probability based on evidence for the connection. The network in Fig 4 only shows interactions with a confidence above 0.9, where the maximum is 1.0. *THRB* is the seed gene from the epistasis network analysis and it is a member of the “RXR and RAR heterodimerization with other nuclear receptor” process. The predicted connection weight between *THRB* and *RXRA* is 0.915. Evidence for this connection includes the following curated by IMP from other sources:

1. *THRB* and *RXRA* are known to participate in the process “RXR and RAR heterodimerization with other nuclear receptor.”
2. BioGRID: Physical interaction between THRB and RXRA
3. Pfam-A, Shared protein domains

THR and vitamin D are known to form heterodimers with RXR, which uses the chromatin assembly process to modulate the transcription of target genes that contain hormone response elements [24]. Using a mammalian two-hybrid assay, Tagami, *et al*., [23] established binding between RXRA and THRB and examined binding among THR mutants. Such experimental data is used in BioGRID. Using the predicted network (Fig 4), a final pathway enrichment analysis was performed (Step 6 of Fig 1, results in Table 1).

**Table 1:** Enriched pathways in the IMP posterior network (Fig 4) with data-driven epistasis network seed genes.

**Biological Process**	**p-value**	**Network Genes**
RXR and RAR heterodimerization with other nuclear receptor	8.48x10^-09^	*RXRA*, *THRA*, *NCOR2*, *THRB*, *PPARA*, *PPARG*
Intracellular receptor mediated signaling pathway	5.10x10^-08^	*RXRA*, *RARA*, *RARG*, *PPARA*, *PPARG*, *ESR1*
retinoic acid receptor signaling pathway	1.38x10^-06^	*RXRA*, *RARA*, *RARG*, *ESR1*
**Functional Annotation**	**p-value**	**Network Genes**
T helper 2 cell differentiation	3.43x10^-11^	*THRA*, *RARA*, *RARG*, *THRB*, *RARB*, *BCL6*
Positive regulation of interleukin-5 production	3.57x10^-10^	*THRA*, *RARA*, *RARG*, *THRB*, *RARB*
Negative regulation of receptor biosynthetic process	3.65x10^-10^	*THRA*, *RARA*, *RARG*, *THRB*, *RARB*, *PPARA*, *PPARG*
RXR and RAR heterodimerization with other nuclear receptor	3.72x10^-10^	*RXRA*, *THRA*, *NCOR2*, *THRB*, *PPARA*, *PPARG*

## Discussion

This study combined a data-driven gene interaction network centrality algorithm with biological pathway knowledge to illuminate the role of RXR in vaccine-induced antibody titer response. To build the data-driven network, we first employed machine learning filters that have been used previously to identify main effect and gene interaction hubs of vaccine response and other phenotypes [5,6]. Simulated evaporative Cooling (EC) aggregates the main and interaction effects for each SNP, and we previously demonstrated the efficacy of EC as a filter for building a gene association interaction network. Unlike typical analyses that consider only the effect of a variant in isolation on a phenotype, SNPrank [6] aggregates the effects of multiple interactions and univariate effects when prioritizing variants. The aggregation of SNP effects with pathway information to amplify the signals of causal variants has been advocated in Ref. [8] Following our epistasis network prioritization, we used pathway enrichment and integration with functional interaction databases to uncover evidence for additional interactions not directly observed in the GWAS.

Previous studies of the retinoid X receptor (RXR) have implicated its role in immunity, particularly through naïve T helper cell differentiation, innate inflammatory response, and cellular apoptosis [25,26]. Notably, multiple studies have revealed associations between genes in RXR-related pathways with variations in differential vaccine immune response [9,11]. Like most complex phenotypes, inter-individual variance in vaccine response involves a number of interacting genes, and any single variant in these genes is unlikely to explain an appreciable proportion of the heritable variation in response [8]. Other mechanisms, including gene-gene interactions (epistasis) and pathway effects (*e*.*g*., RXR), likely account for additional variation and may help identify subtle indirect effects missed by univariate models [5]. Consequently, methods that aggregate effects from multiple gene interactions can help elucidate the genetic architecture of complex phenotypes [6].

A previous study identified an interaction network effect involving a variant in *RXRA* that influenced immune response to smallpox vaccine [5,6]. Although the same variant was not genotyped in the current dataset, the epistasis network analysis uncovered evidence for the involvement of the RXR pathway in inter-individual variation in smallpox vaccine immune response through the effect of RXRA interaction partners. We integrated the epistasis network information with functional pathway information to further characterize genes that may be involved in the smallpox vaccine immune response phenotype, identifying a functional interaction between THRB and RXRA. While imputation can be effective when a potential causal variant is unknown and not tagged in a genotyping array, our results suggest that network integration can uncover important indirect effects in GWAS data, which may be especially important in cases where imputation is less effective. In the current study, we restricted the population to European ancestry to reduce bias due to stratification and to more closely match the population from earlier studies. However, incorporating gene and pathway-level information with GWAS, along with proper ancestry corrections [27], may smooth the effect of combining heterogeneous populations and increase power.

The data-driven portion of the approach led to the enrichment of the pathway “Map kinase inactivation of SMRT co-repressor,” which includes *THRB* and other genes that share biological processes and interactions with *RXRA*. We note that this pathway effect of RXRA is not found when using univariate variant prioritization. The coordinated variation in this biologically relevant pathway provides candidate genes or “seeds” to query the IMP database for additional functional interactions that may be related to the immune response phenotype. The final posterior network implicated RXR pathways involved in vaccine response and included a high-confidence functional interaction between *RXRA* and *THRB*. This final posterior network and enriched pathways reflected the phenotype-specific effects associated with vaccine response while utilizing phenotype-independent biological features.

In addition to RXR pathways, our integrated network analysis implicated “T helper-2 cell differentiation” and “interleukin-5 production,” each of which has been implicated in immune response[28,29], but not specifically to smallpox vaccine response. Moreover, the biological processes uncovered by our pipeline further characterize the role of RXR genes in smallpox vaccine response, which is consistent with our previous smallpox vaccine genetic analysis [5] and other vaccine immune response studies [9,11,25,26]. In summary, our analysis identifies biological pathways previously linked to immune response to the specific (antibody) smallpox vaccine response by seeding data-driven genes in an integrative biological interaction framework. The framework in this methodology applies to other studies wishing to elucidate additional mechanisms that contribute to phenotypic variation on a larger network scale. The filtering steps can be carried out with our command line tools [6], and we plan to simplify integration with IMP in a future implementation of the command line tool using the Sleipnir C++ library [30].

As association studies uncover new genes linked to complex immune responses, such as antibody response to vaccination, a major challenge is to understand how multiple implicated genes work in concert to produce a complex phenotype. Understanding the effects on gene expression–as an intermediate between SNP and phenotype–will lead to more extensive functional models of immune response. For example, in a SNP-SNP interaction analysis of an expression quantitative trait loci (eQTL) study of stimulated and unstimulated cells with the smallpox vaccine, we found a set of genes enriched for apoptosis [31]. Furthermore, *RXRA* has been implicated in the cellular processes leading to apoptosis [32], and the top data-driven pathway in the current study involved SMRT, which involves retinoic acid receptors in the negative regulation of gene expression. Linking these various levels–SNP, expression, methylation and phenotype–may improve our ability to predict and improve immune response to vaccination [33]. Future studies that extend these data sources and algorithms will allow for additional characterization of the biological processes that direct variable response to this and other vaccines. In turn, such information advances the science underlying our understanding of the immunologic effects of immunization with vaccines–a field we have named “vaccinomics” [33,34,35,36,37,38,39,40,41,42]. Such information may be useful in developing novel “next-generation” vaccine candidates, particularly for hyper-variable and complex pathogens where the immune response to the pathogen is correspondingly complex.
